# Supplementary material for: Predictive Performance of Scoring Systems for Mortality Risk in Patients with Cryptococcemia: An Observational Study
Source: J Pers Med. 2023 Sep 6;13(9):1358. doi: 10.3390/jpm13091358 (PMC10533170; doi:10.3390/jpm13091358)
Supplement: Supplementary file 1 [file jpm-13-01358-s001.zip › jpm-2570375-supplementary.pdf]

Supplementary: Table S1

Mortality in emergency department sepsis (MEDS) score

Mortality in emergency department sepsis (MEDS) score	Points
1. Terminal illness with possible death in 1 month	6
2. Hypoxia or tachypnea	3
3. Shock from sepsis	3
4. Platelet count below 150,000	3
5. Granulocytic bands >5% of white blood cells	3
6. Patient older than 65 years old	3
7. Lower respiratory infection	2
8. Patient is from a nursing home	2
9. Mental status is altered	2

Shapiro, N.I.; Wolfe, R.E.; Moore, R.B.; Smith, E.; Burdick, E.; Bates, D.W. Mortality in emergency department sepsis (MEDS) score: a prospectively derived and validated clinical prediction rule. *Crit. Care Med.* **2003**, *31*, 670-675.

Rapid Emergency Medicine Score (REMS)

REMS scoring system	**Score**						
Variable	0	+1	+2	+3	+4	+5	+6
Age (years)	<45		45–54	55–64		65–74	>74
Mean arterial pressure (mm Hg)	70–109		110–129<br>50–69	130–159	>159<br>≤49		
Heart rate (bpm)	70–109		110–139<br>55–69	140–179<br>40–54	>179<br>≤39		
Respiratory rate (breaths/min)	12–24	25–34<br>10–11	6–9	35–49	>49<br>≤5		
O_2 saturation (%)	>89	86–89		75–85	<75		
Glasgow Coma Scale	14 or 15	11–13	8–10	5–7	3 or 4		

Olsson, T.; Terent, A.; Lind, L. Rapid Emergency Medicine Score: a new prognostic tool for in-hospital mortality in non-surgical emergency department patients. *J. Intern. Med.* **2004**, *255*, 579–587.

Modified Early Warning Score

Points	3	2	1	0	1	2	3
Temperature (°C)		35 or less		35.1–38.4		38.5 or higher	
Heart rate (bpm)		39 or less	40–50	51–100	101–110	111–129	130 or higher
Systolic blood pressure (mm Hg)	70 or less	71–80	81–100	101–199		200 or higher	
Respiratory rate		8 or less	9	10–18	19–20	21–29	30 or higher
AVPU score				A	V	P	U

Subbe, C.P.; Kruger, M.; Rutherford, P.; Gemmel, L. Validation of a modified Early Warning Score in medical admissions. *QJM : monthly journal of the Association of Physicians*, **2001**, *94*, 521-526.

Rapid Acute Physiology Score (RAPS) scoring system.

Variable	0	+1	+2	+3	+4
PR (/min)	70–109		55–69	40–54	≤39
			110–139	140–179	≥180
MAP (mmHg)	70–109		50–69	130–159	≤49
			110–129		≥160
RR (/min)	12–24	10–11	6–9	35–49	≤5
		25–34			≥50
GCS	≥14	11–13	8–10	5–7	≤4

PR, pulse rate; MAP, mean arterial pressure; RR, respiratory rate; GCS, Glasgow Coma Scale.

Goodacre, S.; Turner, J.; Nicholl, J. Prediction of mortality among emergency medical admissions. *Emerg. Med. J.* **2006**, *23*, 372–375.

quick sequential organ failure assessment (qSOFA)

	point
Respiratory Rate > 22	1
Systolic Blood Pressure < 100	1
Glasgow Coma Scale <14	1

April, M.D.; Aguirre, J.; Tannenbaum, L.I.; Moore, T.; Pingree, A.; Thaxton, R.E.; Sessions, D.J.; Lantry, J.H. Sepsis clinical criteria in emergency department patients admitted to an intensive care unit: an external validation study of quick Sequential Organ Failure Assessment. *J. Emerg. Med.* **2017**, *52*, 622–631.

National Early Warning Score (NEWS)

**Physiological parameters**	**3**	**2**	**1**	**0**	**1**	**2**	**3**
Respiration Rate (breaths per minute)	≤8		9–11	12–20		21–24	≥25
S_pO_2 (%)	≤91	92–93	94–95	≥96			
Any supplemental oxygen?		Yes		No			
Temperature (°C)	≤35.0		35.1–36.0	36.1–38.0	38.1–39.0	≥39.1	
Systolic BP (mmHg)	≤90	91–100	101–110	111–219			≥220
Heart/pulse rate (beats per minute)	≤40		41–50	51–90	91–110	111–130	≥131
Level of consciousness using the AVPU system				A			V, P or U

Smith, G.B.; Prytherch, D.R.; Meredith, P.; Schmidt, P.E.; Featherstone, P.I. The ability of the National Early Warning Score (NEWS) to discriminate patients at risk of early cardiac arrest, unanticipated intensive care unit admission, and death. *Resuscitation*. 2013, 84, 465-470.

Worthing physiological scoring system

	**Score**			
	**0**	**1**	**2**	**3**
Ventilatory frequency	≤19	20–21	≥22	
Pulse	≤101	≥102		
Systolic blood pressure	≥100		≤99	
Temperature	≥35.3			<35.3
Oxygen saturation in air	96–100	94 to <96	92 to <94	<92
AVPU	Alert			Other

Duckitt, R.W.; Buxton-Thomas, R.; Walker, J.; Cheek, E.; Bewick, V.; Venn, R.; Forni, L.G. Worthing physiological scoring system: derivation and validation of a physiological early-warning system for medical admissions. An observational, population-based single-centre study. *Br. J. Anaesth*. **2007**, *98*, 769-774.

Glasgow coma scale, age, and arterial blood pressure score

Variable/score	0	+1	+3	+4	+6
Glasgow Coma Scale		1 points per GCS			
Age	≥60		<60		
Systolic blood pressure	<60			60-120	>120

Higher points denotes better outcome in this case, out of a possible 24 points.
